# Metagenomic insight into the biodegradation of biomass and alkaloids in the aging process of cigar

**DOI:** 10.1186/s40643-023-00667-y

**Published:** 2023-07-24

**Authors:** Fang Xue, Juan Yang, Cheng Luo, Dongliang Li, Guiyang Shi, Guangfu Song, Youran Li

**Affiliations:** 1 Key Laboratory of Chinese Cigar Fermentation, Cigar Technology Innovation Center of China Tobacco, China Tobacco Sichuan Industrial Co., Ltd, Chengdu, 610000 China; 2 National Engineering Research Center for Cereal Fermentation and Food Biomanufacturing, Jiangnan University, 1800 Lihu Avenue, Wuxi, 214122 Jiangsu People’s Republic of China; 3 Jiangsu Provincial Engineering Research Center for Bioactive Product Processing, Jiangnan University, 1800 Lihu Avenue, Wuxi, 214122 Jiangsu People’s Republic of China

**Keywords:** Cigar, Natural aging, Microbial community, Environmental factors

## Abstract

A significant distinction between cigar production and tobacco lies in the necessary aging process, where intricate microbial growth, metabolic activities, enzymatic catalysis, and chemical reactions interact. Despite its crucial role in determining the final quality of cigars, our comprehension of the underlying chemical and biological mechanisms within this process remains insufficient. Biomass and alkaloids are the primary constituents that influence the flavor of cigars. Consequently, investigating the entire aging process could begin by exploring the involvement of microbes and enzymes in their biodegradation. In this study, handmade cigars were aged under different conditions. Metagenomic sequencing was employed to identify the microbes and enzymes responsible for the degradation of biomass and alkaloids derived from tobacco leaves. The results revealed that various environmental factors, including temperature, humidity, duration time, and turning frequency, yielded varying contents of total sugar and alkaloids in the cigars. Significant correlations were observed between microbial communities and starch, reducing sugars, total sugars, and alkaloids. Key species involved in the breakdown of biomass constituents, such as starch (*Bacillus pumilus*, *Pseudomonas* sp. 286, and *Aspergillus cristatus*), reducing sugars and total sugars (*Aspergillus cristatus* and *Nitrolancea hollandica*), were identified. Furthermore, *Corynespora cassiicola* and *Pseudomonas fulva* were found to potentially contribute to the degradation of alkaloid compounds, specifically nornicotine and neonicotinoid. Our work contributes to a deeper understanding of the microbial roles in the aging of cigars. Moreover, the selection of specific microbial strains or starter cultures can be employed to control and manipulate the aging process, thereby further refining the flavor development in cigar products.

**Graphical Abstract:**

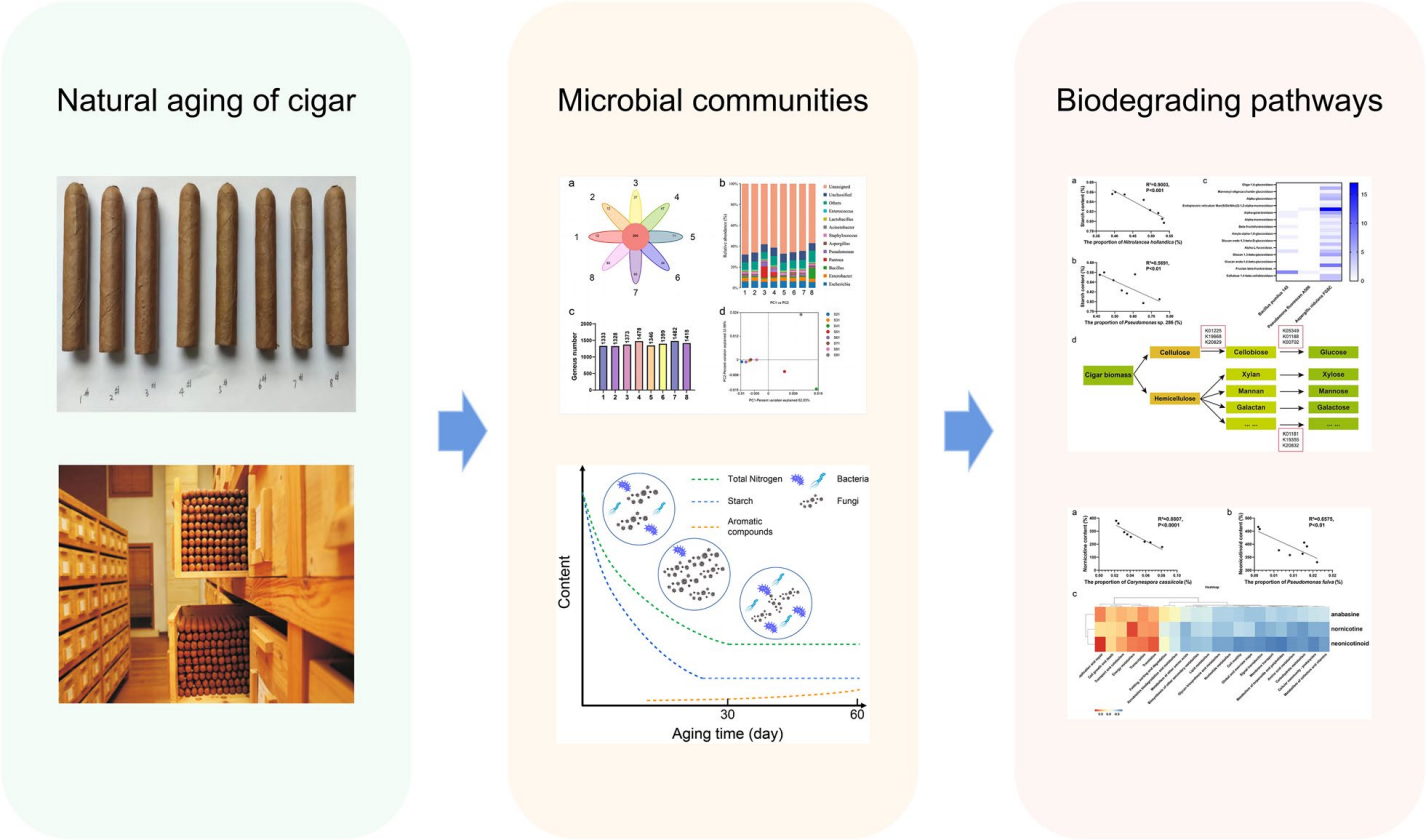

## Introduction

Cigars are distinctive tobacco products composed entirely of tobacco leaves, renowned for their pronounced aroma, rich flavor, and robust alkaline smoke. Notably, cigars exhibit a low tar-to-nicotine ratio (Wanrong et al. 2022; Wen et al. 2022). The production process of cigar mainly includes agricultural fermentation of tobacco leaves, industrial fermentation, rolling and aging. Subsequent to fermentation and rolling, the cigars undergo a final aging process. This involves storing the cigars in carefully controlled conditions, such as wooden humidors or aging rooms. Since the aging environment is relatively mild, a large number of enzymes and microorganisms are retained and active. They can continue to interact with the chemical components of tobacco leaves during the aging process, ultimately determining the finished quality of a cigar (Hu et al. 2022). Specifically, the aging process allows for the breakdown of harsh compounds, the melding of flavors, and the enhancement of overall smoothness (Hu et al. 2023; Zheng et al. 2022).

One of a significant influence on cigar aging is biodegradation of biomass and alkaloids. It is mainly because that biomass and alkaloids are the primary organic compounds found in cigar leaves. In flue-cured tobacco leaves, biomass contents fall within the ranges of 20–50%, including starch, cellulose, hemicellulose and reducing sugars (Zhao et al. 2022). These carbohydrates compounds can be essential sources of nutrition for microorganisms. Meanwhile, microbes break down these components into simpler compounds through enzymatic activity, leading to the formation of various flavor compounds. These compounds include aldehydes, ketones, sulfur compounds, and various other volatile organic compounds. The specific microbial community present during fermentation influences the composition and concentrations of these compounds, thereby impacting the final flavor profile of the cigar (Zhang et al. 2020). For instance, *Bacillus* and *Aspergillus* can convert sugars into organic acids, alcohols, and esters, which contribute to the overall flavor profile of fermented tobacco. These compounds can impart fruity, floral, or wine-like notes, enhancing the complexity and character of the tobacco flavor (Krusemann et al. 2018). Alkaloids, on the other hand, are a specific type of chemical compound that are often responsible for the psychoactive effects. Some of the most common alkaloids found in cigar leaves include nicotine, anabasine, and nornicotine (Hu et al. 2023). Microbial degradation helps in removing undesirable compounds from tobacco leaves during aging. For example, some microorganisms can metabolize or modify bitter alkaloids present in the leaves. This detoxification process can lead to a milder and smoother taste, reducing the harshness associated with raw tobacco (Hartmann 2007; Paiva 2000).

During cigar aging, different microbial species and strains succeed each other over time, creating a dynamic microbial community. The interactions between these microorganisms, including competition, cooperation, and metabolic cross-feeding, influence the aging process and the resulting flavor. This microbial succession can lead to the production of unique flavor compounds that define the characteristics of specific tobacco varieties or fermentation techniques. It is important to note that cigar aging process is a complex biological process influenced by multiple factors, such as temperature, moisture content and duration time. Cigars are typically aged at a controlled temperature, usually between 16 °C and 20 °C, to promote development of desired characteristics and control the the risk of tobacco beetle infestations. The ideal humidity level for aging cigars is generally maintained between 60% and 70% relative humidity. This range helps prevent the cigars from becoming too dry or overly moist, preserving the tobacco's flavors and preventing mold growth. The duration time of cigar aging typically range from several months to several years. Longer aging periods allow for more complex chemical reactions to occur, resulting in enhanced flavor development and improved overall quality. Careful management of these factors is essential to achieve desired flavor profiles and consistency in cigar production. The mechanism of interaction between the above factors during the aging of the cigar has not been studied and only speculations have been made. For example, since the preparation environment of cigar tobacco leaves is relatively mild, a large number of active enzymes and microorganisms are retained after rolling (Hui-yuan et al. 2022; Yao et al. 2022). They can continue to interact with the chemical components of tobacco leaves during the aging process. Based on the high throughput screening (HTS) methods on the diversity of functional tobacco microbial communities, the dominant microbial communities identified in the cigar fermentation process in countries around the world include *Bacillus*, *Staphylococcus*, *Debaryomyces hansenii*, *Jeotgalicoccus*, *Lactobacillus*, *Weissella*, *Corynebacterium*, *Yaniaccac*, *Pseudomonas*, and *Acinetobacter* (Zhang et al. 2020; Zhao et al. 2007; Zhou et al. 2021). Understanding the functional microorganisms in cigar leaves is the basis for analyzing the aging mechanism. Existing studies have yielded some understanding of the structural characteristics and functions of microbial communities in the fermentation process of various types of tobacco leaves, but knowledge is still limited concerning the microorganisms and their functions during the aging process of cigars (Hu et al. 2022; Liu et al. 2021, 2022).

This study designed cigar aging experiments with different conditions and factors. HTS at a metagenomics level was used to characterize dynamic changes in the sample microbial communities, on top of which association analysis is conducted regarding the successions of microbial communities and the main chemical components of cigars. Relationship between the degradation biomass and alkaloids of tobacco leaves and microbial communities under different aging conditions was investigated. The results can provide a scientific basis and guidance for the analysis and regulation of the aging of cigars. In addition, the selection of specific microbial strains or starter cultures can be employed to control and manipulate the aging process, further fine-tuning the flavor development in cigar products.

## Materials and methods

### Cigar sample collection

The cigar samples used in this study were made with Luzhou Sun-cured tobacco and were stored in the cigar aging warehouse of Sichuan China Tobacco Industry Co., Ltd., Great Wall Cigar Factory, after being rolled and wrapped in cigar jackets. All the samples were received in January 2022, with a moisture content of 17% ± 1% upon arrival. The warehouse environment maintained a humidity of 70% ± 5% and a temperature range of 18–27 °C. The aging process took place in a cabinet made of Spanish pine wood. The aging conditions were set based on the existing cigar aging process, including temperature, humidity, duration time, and turning frequency, as shown in Table 1. Under each experimental condition, 20 cigars were aged, and 2 of them were randomly selected as parallel samples for microbiome testing and chemical composition analysis. Sample information, sampling time, and sampling location are also recorded. Samples are stored in a sterile sampling bag at −80 °C until removed for testing.

**Table 1:** Aging conditions for different samples

Sample no	Temperature (℃)	Humidity (%)	Time (d)	Turning frequency (d/times)
1	20	65	60	30
2	20	65	90	30
3	16	65	90	30
4	24	65	90	30
5	20	60	90	30
6	20	70	90	30
7	20	65	90	45
8	20	65	90	90

### Sample processing, DNA extraction and metagenome sequencing

About 2 g cigar samples were placed into 250 mL shake flasks containing 50 mL of sterile 1 × phosphate-buffered saline (PBS) buffer. Microbes on fermented leaves were collected by shaking the flasks and centrifuging the eluent. Metagenomic DNA was isolated using a soil genome extraction kit E.Z.N.A. (Omega, USA) following the manufacturer’s instructions. ND-1000 UV–Vis ultraviolet spectrophotometer (Thermo Scientific NanoDrop, USA) and gel electrophoresis were used to assess DNA concentration and quality. High-quality DNA samples were submitted to the Biomarker Co., Ltd. (Beijing, China) for paired-end meta-genomic sequencing. Sequencing libraries were constructed using 1 μg of each metagenomic DNA sample with ~ 300 bp fragments. High-throughput sequencing was performed on the Il-lumina HiSeq4000 platform (Illumina, San Diego, CA, USA) using the paired-end 150 bp sequencing strategy.

### Cigar chemical composition analysis

A halogen moisture analyzer (DHS-10A, LICHEN, CHINA) is used to measure the moisture content of the cigar, and the mass of the samples before and after drying through the moisture analyzer is recorded separately. The difference between the two masses divided by the mass of the fresh sample is the moisture content of the sample. A cyclone mill is used to grind all samples to powders. According to standards YC/T 216–2013, YC/T 249–2008, and YC/T 159–2002 of the Chinese tobacco industry, starch, protein, and soluble sugar contents were extracted and measured by a Pulse 3000 flow injection analyzer (Systea, Italy). In summary, the cigar samples underwent a series of extraction and analysis processes. Initially, a 30-min ultrasonic extraction using an 80% ethanol-saturated sodium chloride solution was employed to eliminate interfering substances. Subsequently, the extraction solution was discarded, and a 10-min ultrasound extraction was conducted using a 40% concentrated perchloric acid solution. For starch analysis, a color reaction with iodine under acidic conditions occurred, and the absorbance was measured at 570 nm. In the case of protein analysis, the protein was subjected to intense heat digestion in the presence of concentrated sulfuric acid and a catalyst, resulting in the conversion of nitrogen into ammonia. Under alkaline conditions, ammonia was oxidized to ammonium chloride by sodium hypochlorite and then reacted with sodium salicylate to produce a dye. The absorbance was measured at 660 nm. To detect soluble sugar components, a 5% acetic acid aqueous solution was used for extraction. The sugars in the extraction solution reacted with p-hydroxybenzoic acid hydrazide, leading to the generation of a yellow azo compound in an alkaline medium at a temperature of 85 °C. The absorbance was measured at 410 nm. The content of free amino acids and organic acids in the cigar is measured using Li's method (Li et al. 2011). Briefly, a solution containing water, methanol, and acetonitrile in a volume ratio of 3:1:1 was employed for the extraction of cigar powder. The resulting suspension underwent sonication and centrifugation to collect the supernatant. Subsequently, freeze-drying was conducted on the collected supernatant. Methoxyamination and trimethylsilylation procedures were then carried out on the freeze-dried samples. Finally, N-methyl-N-(trimethylsilyl) trifluoroacetamide was added to the mixture and incubated before subjecting it to GC–MS analysis.

### Statistical analysis

Raw sequence reads which contained sequencing adapters (overlap > 15 bp) and low-quality reads (*Q* value < 38) were trimmed by Trimmomatic v0.36. The trimmed reads were aligned to the genomes of *Nicotiana tabacum*, *Nicotiana sylvestris* and *Nicotiana tomentosiformis* using bowtie (Langmead and Salzberg 2012), and the corresponding mapped reads were removed (Kang et al. 2019). The remaining clean reads were assembled by the SOAPdenovo2 (Luo et al. 2012) with a k-mer length of 39–47. Open Reading Frames (ORFs) were predicted from assembled contigs (> 100 bp) using MetaGeneMark (Noguchi et al. 2006) with default parameters. Taxonomic annotation of predicated genes in each sample was obtained using MetaPhlAn2 (Truong et al. 2015). Functional annotation of predicted genes was performed by BLASTP against eggNOG database (Huerta-Cepas et al. 2019) and by GhostKOALA 1.0 (Kanehisa et al. 2016) against Kyoto Encyclopedia of Genes and the Genomes (KEGG) database. Genetic sequences obtained are processed using QIIME 2 (Bolyen et al. 2019). Simply put, raw sequencing readings are assigned to specific samples using exact matches to barcode sequences and filtered to exclude low-quality sequences, namely, those with < 150 bp in length, mean Phred score < 20, ambiguous bases, and/or single nucleotide repetition > 8 bp. The remaining high-quality paired-end readings are assembled using FLASH (Magoc and Salzberg 2011). After the detection and removal of chimera, the remaining high-quality sequences are clustered into amplified sequence variants. Classification is performed using the Q2 feature classifier QIIME 2 plug-in to implement the sklearn method and the pre-trained SILVA database (version 132) (Quast et al. 2013), with a similarity of 99%.

Species diversity matrices are presented based on the binary Jaccard index, and principal component analysis (PCA), multiple comparisons, and heat mapping are performed using the R language platform (v.4.0.0). To determine different taxa between two groups, the linear discriminate analysis (LDA) effect size (LEfSe) algorithm on the Galaxy browser (https://huttenhower.sph-harvard.edu/galaxy/) was used (Segata et al. 2011). Furthermore, based on Spearman's rank correlation coefficient (p < 0.05, |r|> 0.3), network analysis is performed using Gephi on the correlation between representative microorganisms and core chemical components. For bacterial and fungal communities, the large sequence libraries are classified with ecological significance using PICRUSt2 (Douglas et al. 2020) and FUNGuild (Nguyen et al. 2016). All data are standardized during the statistical analysis.

## Results

### The content of organic compounds in cigar leaves under different aging conditions

**Fig. 1:**
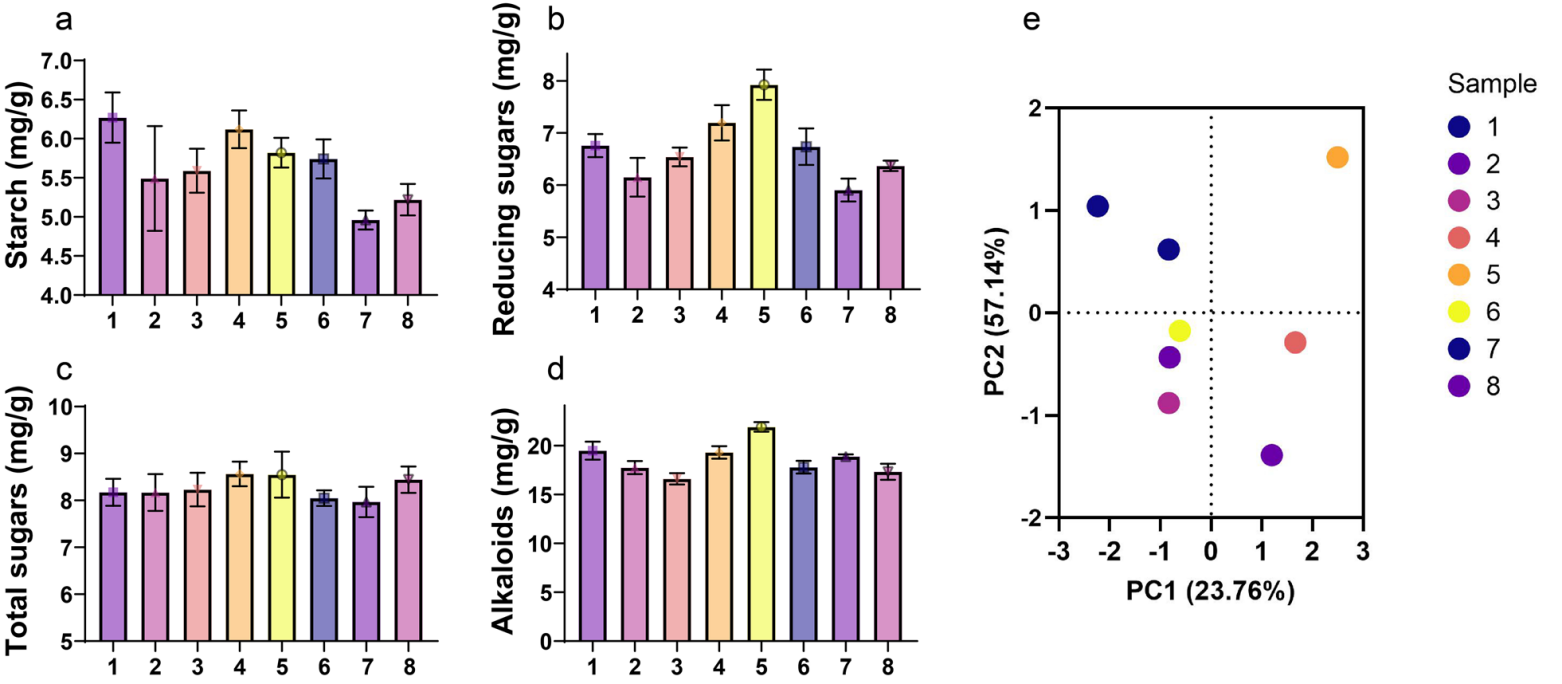
Contents of starch (**a**), reducing sugars (**b**), total sugars (**c**) and alkaloids (**d**) of different samples. PCA plot showing compounds distribution of different samples (**e**). The sample numbers corresponded to the samples depicted in Table 1

The contents of four main organic compounds, namely, starch, reducing sugars, total sugars and alkaloids, were analyzed and presented in Fig. 1. Aging time significantly affects the starch, reducing sugars and total sugars content of cigars. During a 90-day aging process, the starch content of cigar leaves shows a trend of rapid decline followed by a gradual decrease with increasing aging time, while the content of reducing sugars and total sugars shows a trend of rapid increase followed by a gradual decrease. Specifically, the starch and total sugars in sample 2 (90 days) were significantly (*P* < 0.05) lower than those in sample 1 (60 days). Alkaloids exhibit little fluctuation during the aging process. The change in starch content indicates the participation of microorganisms during the maintenance process, which further degrades the large molecular compounds such as starch in cigar leaves into reducing sugars. This aligns with people's expectations, as it is widely believed that the presence of reducing sugar positively impacts tobacco-smoking characteristics, enhancing both flavor and aroma (Banozic et al. 2020). Aging temperature has a significant effect on the starch and alkaloids content of cigars, with the alkaloids showing an increasing trend. The content of reducing sugars and total sugars shows little fluctuation under different temperature conditions. Humidity has a significant effect on the reducing sugars and alkaloids of cigars, with the alkaloids being the most affected. With an increase in aging humidity, the reducing sugars and alkaloids content of 90-day-aged cigars show a decreasing trend, while the starch and total sugars content exhibit minimal fluctuation. It can be proposed that an increase in aging humidity is beneficial to the degradation of alkaloids. In summary, achieving a well-balanced composition of starch, sugar, and alkaloids is crucial for the quality of cigars. Appropriate levels of these components contribute to the desired flavors, aromas, and overall smoking experience. However, excessive amounts can result in undesirable flavors, harshness, stickiness, or discomfort.

### Taxonomy annotation of microbial communities in cigar

**Fig. 2:**
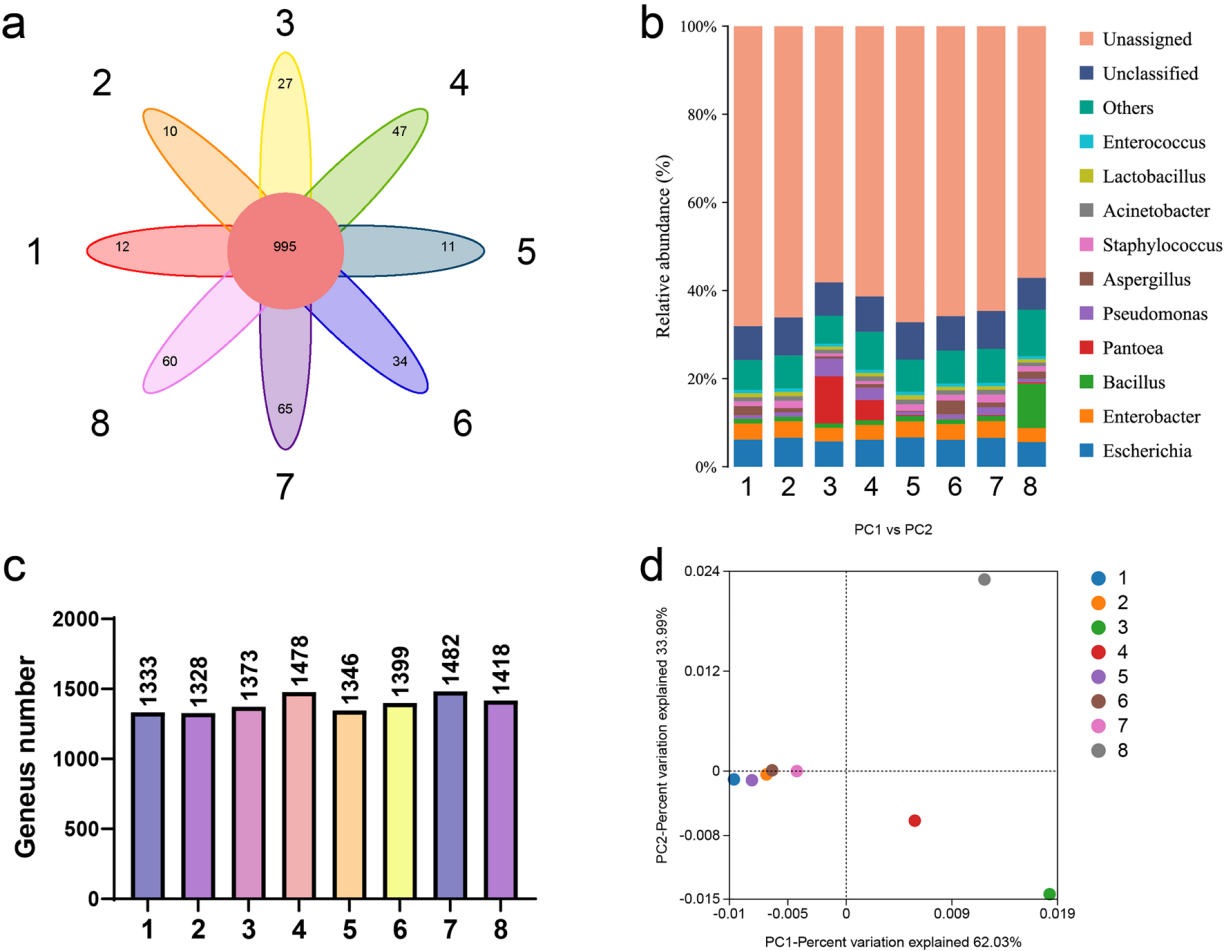
Microbial community structures of aging cigar at the genus level. **a**, **c** Venn diagram depicting number of genera identified in cigar microbiomes under different aging conditions. **b** Stacked bar chart showing genus composition of each sample based on relative abundance data. **c** PCA plot showing composition similarity between samples at the genus level. The sample numbers corresponded to the samples depicted in Table 1

The metagenome of cigar samples was analyzed and the microbial communities present were identified. To remove host-originated reads, we aligned the metagenome reads to the draft genomes of *Nicotiana tabacum*, *Nicotiana sylvestris*, and *Nicotiana tomentosiformis*. This resulted in 632,073,854 paired-end clean reads that were used for de novo assembly. We predicted 5,276,333 open reading frames (ORFs) with an average length ranging from 102 to 14,595 bp, and an average length of 244 bp to 291 bp. Our analysis revealed that bacteria were the dominant members, ranging from 83.5% to 92.4% in cigar samples, with fungi being the second most prevalent members, ranging from 6.0% to 16.1%. Archaea and viruses were observed at low abundance. At the genus level, there were 995 genera shared by all communities (Fig. 2a, c). Among the samples analyzed, the one subjected to a humidity of 60% during aging displayed the lowest overall diversity of genera. Conversely, the sample aged for 90 days and subjected to the lowest frequency of turning demonstrated the highest diversity of genera. Furthermore, a notable finding was the significant variation in the dominant genus members when the aging temperature was altered from 20 °C to either 16 °C or 24 °C (Fig. 2b). The genera composition on PCA plot showed three clusters among the eight communities (Fig. 2d). *Pantoea* was prevalent genus in samples 3 and 4, *Escherichia* was the most abundant genus in samples 1, 2, 4, 5, 6, and 7. Sample 8 was predominately composed of *Bacillus*.

### Redundancy analysis between aging conditions and microbial communities

**Fig. 3:**
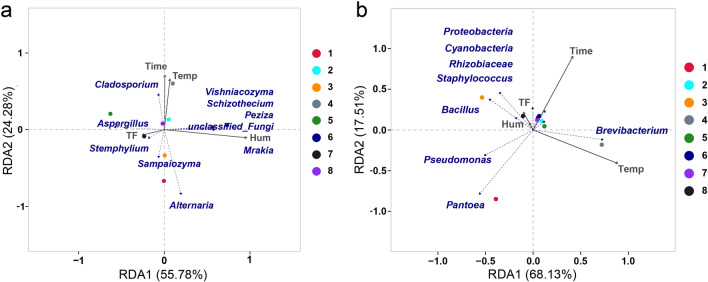
Distribution characteristics of microbial communities at the genus level in the cigar samples. RDA biplot showing the correlation between fungal communities and different aging conditions (**a**). RDA biplot showing the correlation between bacterial communities and different aging conditions (**b**). The sample numbers corresponded to the samples depicted in Table 1

RDA was performed to investigate potential associations between aging conditions and the microbial community structure, specifically focusing on the top 10 taxonomic groups at the genus levels (Fig. 3). Figure 3a illustrates a positive correlation between humidity and the abundance of *Peziza*, *Mrakia*, *Vishniacozyma* and *Schizothecium*. The *X*-axis accounts for 55.78% of the variation at the genus level for fungi, positively correlating with humidity and negatively correlating with turning frequency. In addition, the *Y*-axis explains an additional 24.28% of the variation, positively correlating with time and temperature. Turning frequency, identified as a common influencing factor for both axes, significantly impacts the microbial community structure in the aging samples of cigars. This influence is exerted through modulating the availability of oxygen, thereby shaping the composition, diversity, and metabolic potential of microbial populations. A smaller angle between the arrows associated with the top 10 core taxa and groundwater parameters indicates a stronger correlation. The RDA biplot indicates that humidity and turning frequency play critical roles in the composition of fungal microbial communities. Contrasting with most other fungi, *Aspergillus*, the predominant fungal population during aging, exhibits divergent responses to the four environmental conditions. Specifically, *Aspergillus* demonstrates a positive correlation with turning frequency and a negative correlation with humidity. In contrast, the majority of other fungi display the opposite pattern. Similar analysis was conducted for bacterial microbial communities, as depicted in Fig. 3b, which also highlight the dominance of humidity and turning frequency. The *X*-axis accounts for 68.13% of the variation at the genus level, positively correlating with temperature and aging time. Furthermore, the *Y*-axis explains an additional 17.51% of the variation, positively correlating with humidity and turning frequency. While most bacterial genera exhibit positive correlations with humidity and turning frequency, *Brevibacterium* demonstrates a positive correlation with aging temperature.

### Relationship between chemical components of tobacco leaves and microbial communities

**Fig. 4:**
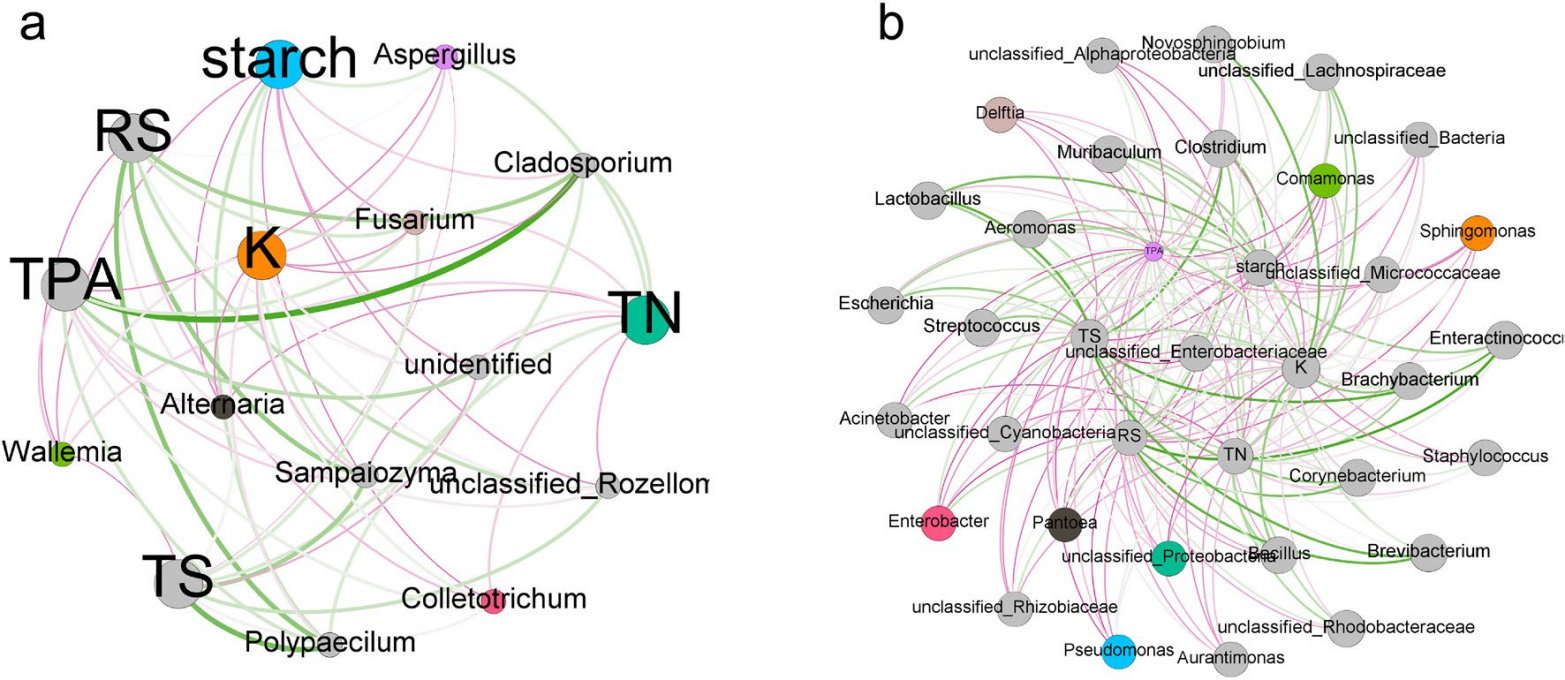
Network diagram of a correlation between conventional components of tobacco leaves and fungal (**a**) and bacterial (**b**) microbial communities under different aging conditions. The green curve indicates a positive correlation and the red curve indicates a negative correlation

The biodegradation process of starch, total nitrogen, reducing sugars and total sugars during the aging of cigars is closely linked to the development of cigar properties, particularly the taste and aroma of the smoke. Different conditions result in a reduction in starch content and an increase in reduced sugar content after aging. We also analyzed the content of each component in relation to the distribution of microbial communities. The results revealed a strong correlation between two components, starch and total nitrogen, and fungal communities, while no significant correlation was observed between the content of reducing sugars, total sugars, and either fungal or bacterial communities. Figure 4a demonstrates that starch is negatively correlated with major fungal communities, indicating that the decrease in starch content during aging is primarily attributed to the activity of fungal communities. Previous studies by Liu et al. (2021) investigated the microbial diversity of cigar tobacco at different fermentation stages and found that *Aspergillus* is the dominant group before aging. Considering that *Aspergillus* possesses a strong ability to decompose starch and organic nitrogen under conditions with sufficient oxygen and appropriate water activity (Yang et al. 2022), we propose the following mechanism for starch biodegradation during cigar aging: during the initial stage of aging, *Aspergillus* takes advantage of the relatively abundant starch and organic nitrogen in tobacco leaves to proliferate and establish dominance. During the first 30 days of the aging process, the *Aspergillus* community exhibits a high rate of starch decomposition, which gradually decreases as other microbial communities begin to contribute significantly to starch degradation. In addition, Fig. 4b illustrates a negative correlation between starch and dominant bacterial communities, such as *Pseudomonas*, *Bacillus*, and *Enterobacter*. These bacteria are capable of secreting starch hydrolytic enzymes and efficiently metabolizing reducing sugars. Notably, a key distinction between bacteria and fungi lies in the higher number of fungal communities capable of degrading high molecular weight proteins. This is evident in the significantly higher abundance of the fungal community compared to the bacterial community, which also shows a strong negative correlation with total nitrogen content.

### Metagenomic annotations and functional profiles of microbial communities in cigar

**Fig. 5:**
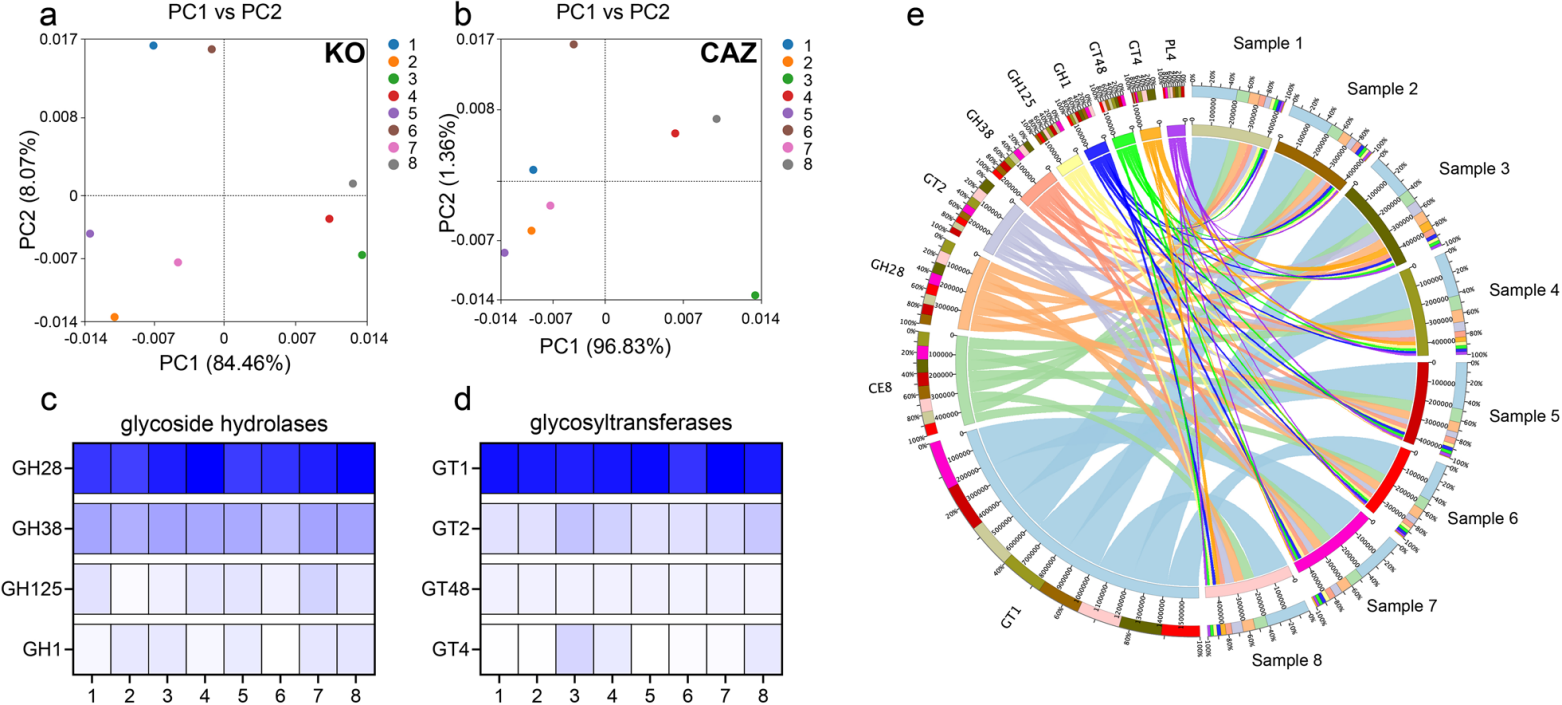
Functional distribution and diversity of microbial communities between different samples based on KO, and CAZ functional profiles. **a**, **b** PCA plots of functional genes of KO and CAZ showing significant differences between samples. **c**, **d** Heatmap of the relative abundance of the CAZy relating to glycoside hydrolases and glycosyltransferases among samples. **e** Functional categories of different samples

Metagenomic annotations were employed to explore the functional diversity and biopolymer-degrading capabilities of microbial consortia. The analysis revealed that 97.42% of the predicted genes corresponded to coding DNA sequences (CDSs), with 68.72% of CDSs assigned putative functions through database comparisons with eggNOG and KEGG. KEGG ontology analysis highlighted energy metabolism, translation, carbohydrate metabolism, and amino acid metabolism as the most abundant metabolic functions. Principal Component Analysis (PCA) based on lower functional units of KO and CAZ was conducted to assess the functional composition of different groups (Fig. 5a, b). The results indicated significant differences between sample 4 and 8 compared to the other three groups, while sample 6 exhibited separation from samples 1, 2, 5, and 7 in terms of KO and CAZ profiles. Samples 3, 4, and 8 exhibited higher functional diversity, while sample 5 displayed relatively lower diversity of KO and CAZ compared to other samples. Starch and cellulose, composed of monosaccharides linked by glycosidic bonds, were identified as the primary biopolymers. Enzymes involved in the biodegradation of starch and cellulose were mainly classified into four Glycoside hydrolase (GH) families (GH1, GH25, GH28, and GH38) and four glycosyltransferase (GT) families (GT1, GT2, GT4, and GT48). The distribution of CAZymes across the samples indicated that GH28 and GT1 were the most abundant families. Furthermore, the analysis of GH family enzymes in the samples (Fig. 5c) revealed that sample 6 exhibited the lowest hydrolase activity for starch and cellulose, suggesting a weak biodegradation capacity under high humidity. The distribution of GT family enzymes (Fig. 5d) indicated that sample 3 possessed the highest glycosyltransferase content, suggesting a strong biotransformation capacity under low temperature. To further explore the biodegradation capacities, genes encoding all carbohydrate-active enzymes were obtained and categorized from the metagenomic data, with GHs and GTs identified as the primary enzymes in all samples (Fig. 5e). In summary, these findings provide valuable insights into the functional diversity and biopolymer-degrading capacities of microbial consortia.

**Fig. 6:**
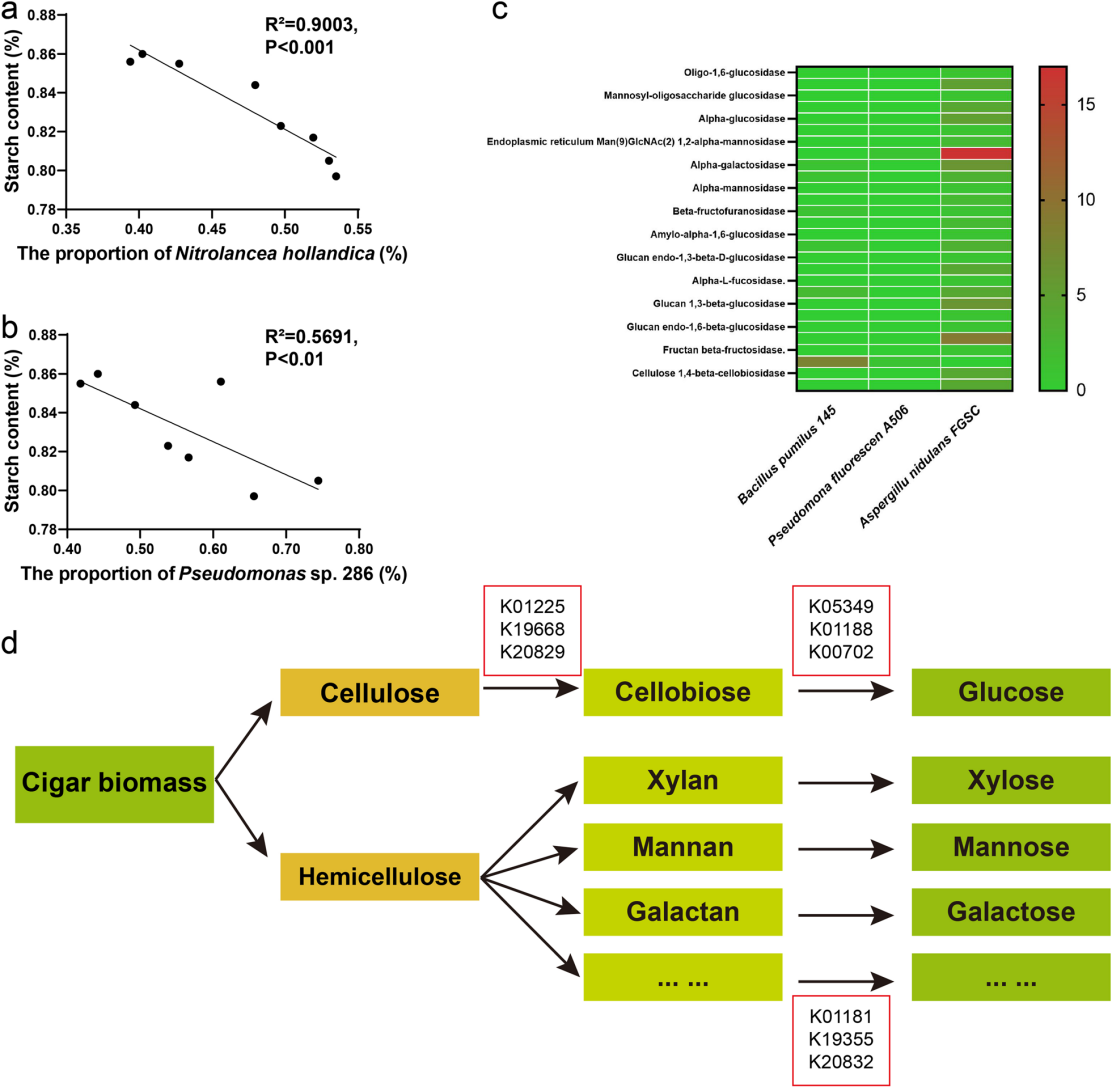
Correlations between vital species and carbohydrates in cigar: the relative abundance of *Nitrolancea hollandica* (**a**) and *Pseudomonas* sp. 286 (**b**) with starch content. Comparison of the relative abundance of genes relating to carbohydrate degradation among vital species (**c**). The metabolic pathway relating to cigar biomass catabolism from the vital species (**d**)

### Mining for important microbes and metabolic pathways from metagenome

Correlation analysis were used to examine the relationship between main chemical compounds and dominant microbial communities. The results showed that there were 10, 9 and 10 genera significantly (*P* < 0.05) correlated with starch, reducing sugars and total sugars, respectively (Table 2). *Bacillus* was negatively correlated with starch and reducing sugars. *Aspergillus* was negatively correlated with starch, reducing sugars and total sugars. The negative correlations between key species and related compounds indicated their important roles in the degradation of natural compounds in cigar. It was suggested that vital species in the catabolism of starch (*Bacillus pumilus*, *Pseudomonas* sp. 286 and *Aspergillus cristatus*), reducing sugars and total sugars (*Aspergillus cristatus* and *Nitrolancea hollandica*) were distributed among different genera (Fig. 6a, b). The genomic information of the typical strains (*Bacillus pumilus* 145, *Pseudomonas fluorescens* A506 and *Aspergillus nidulans* FGSC) were obtained from IMG database (https://img.jgi.doe.gov/) (Fig. 6c). The function annotation of the three strains further indicated more carbohydrate degrading enzymes (such as α-galactosidase, α-mannosidase, 6-phospho-β-glucosidase, β-glucosidase, xylan 1,4-β-xylosidase, glucan 1,3-β-glucosidase and non-reducing end α-L-arabinofuranosidase) (Fig. 6d).

**Table 2:** Dominant genera (relative abundance > 1%) correlating with the contents of starch, reducing sugars, total sugars and alkaloids

Dominant genera	Starch		Reducing sugars		Total sugars		Alkaloids	
	*r*	*p*	*r*	*p*	*r*	*p*	*r*	*p*
*Escherichia*	0.381	0.031	0.333	0.038	0.238	0.071	0.667	< 0.001
*Enterobacter*	0.048	0.038	−0.071	0.041	−0.095	0.039	0.500	< 0.001
*Bacillus*	−0.681	< 0.001	−0.767	< 0.001	0.048	0.231	0.333	0.030
*Pantoea*	−0.143	0.073	0.143	0.130	0.071	0.043	−0.262	0.062
*Pseudomonas*	0.000	0.032	−0.119	0.055	−0.143	0.058	−0.524	0.152
*Aspergillus*	−0.805	< 0.001	−0.571	< 0.001	−0.548	< 0.001	−0.095	0.234
*Staphylococcus*	−0.048	0.079	−0.071	0.070	−0.119	0.031	0.214	0.070
*Acinetobacter*	0.624	0.001	0.524	0.001	0.310	0.034	0.571	0.065
*Lactobacillus*	0.214	0.176	0.190	0.046	0.071	0.039	0.786	< 0.001
*Enterococcus*	0.429	0.048	0.286	0.034	0.286	0.050	0.548	0.001
*Klebsiella*	0.719	< 0.001	0.310	0.252	0.262	0.130	0.214	0.138
*Sinorhizobium*	0.024	0.036	0.024	0.048	0.167	0.214	0.476	0.537
*Acetobacter*	0.310	0.079	0.143	0.057	-0.190	0.048	0.262	0.333
*Rhizophagus*	0.524	0.070	0.524	0.001	0.452	0.001	0.214	0.445
*Paenarthrobacter*	0.357	0.041	0.238	0.033	0.071	0.250	0.310	0.143

^*^r Relative index, *p* Statistical significance

**Fig. 7:**
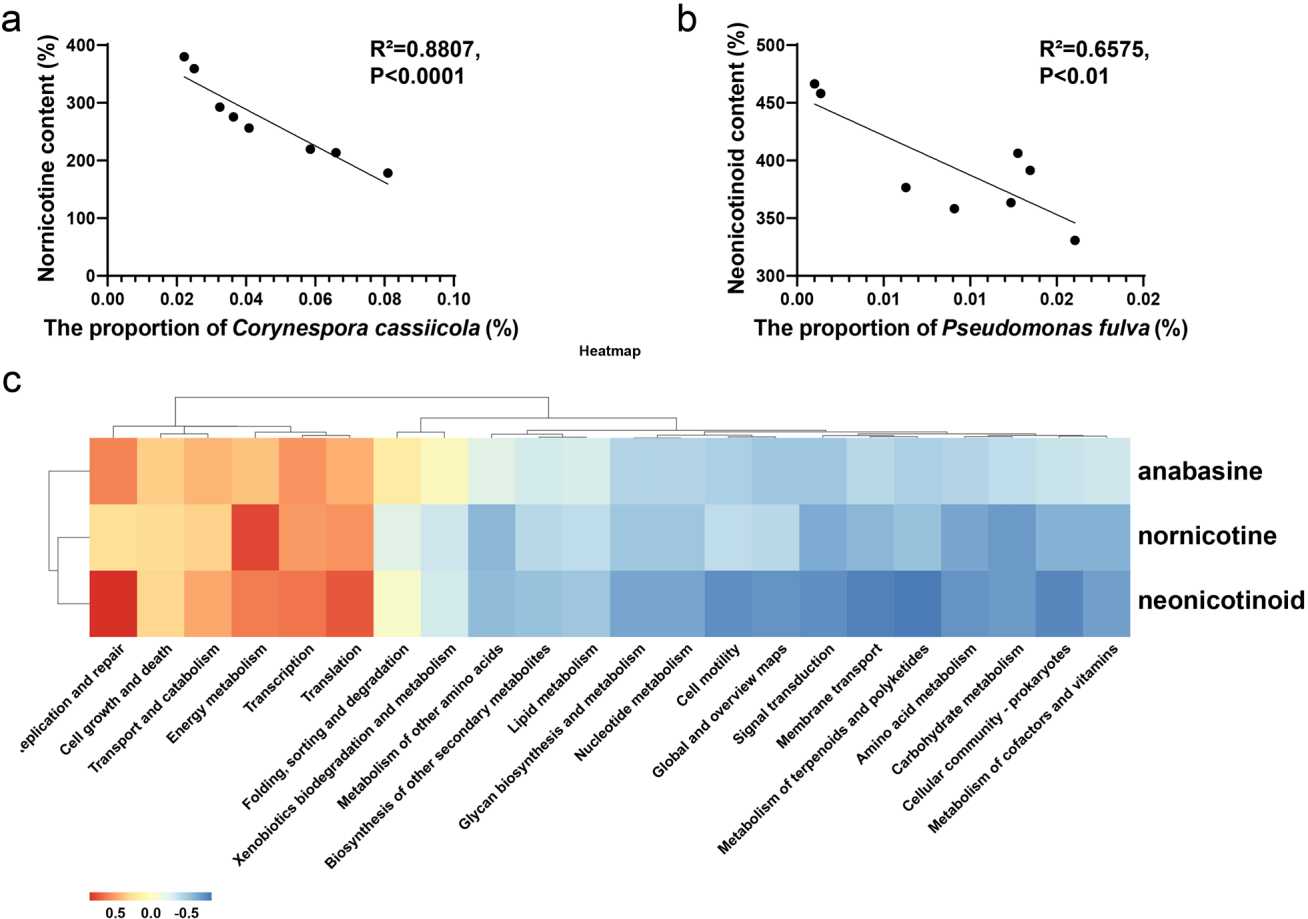
Correlations between vital species and alkaloids in cigar: the relative abundance of *Corynespora cassiicola* (**a**) and *Pseudomonas fulva* (**b**) with nornicotine and neonicotinoid, respectively. Comparison of the relative abundance of genes relating to anabasin, nornicotin and neonicotinoid degradation among vital species (**c**)

By comparing the magnitude of the correlation coefficient between microbial genera and alkaloids, it was found that *Corynespora* and *Pseudomonas* were negatively correlated with different alkaloids, such as nornicotine and neonicotinoid (Fig. 7). The numbers of microbial species associated with these alkaloids were significantly smaller than those of starch, reducing sugars or total sugars. *Corynespora cassiicola* and *Pseudomonas fulva* were proposed to contribute to the degradation of nornicotine and neonicotinoid, respectively, as they had significantly negative correlation (*P* < 0.05) with the chemicals. Microorganisms from the genera of *Pseudomonas* were comprehensively reported for their capability of degrading nicotine through the pyrrolidine pathway. The pyrrolidine ring is first dehydrogenated to N-methylmyosmine, followed by spontaneous hydrolysis to form pseudooxynicotine that is then oxidized to 3-succinoylpyr-idine. Two in four genes (2,5-DHP dioxygenase gene, N-formylmaleamate deformylase gene, maleamate amidase gene and maleate cis–trans isomerasegene) reported to be involved in this process could be identified in the genome of *Pseudomonas fulva*. The correlation between metabolic pathways and typical alkaloids, anabasin, nornicotin and neonicotinoid, was analyzed. Results showed that metabolism of terpenoids and polyketides and metabolism of cofactors and vitamins were main pathways negatively correlated with the alkaloids. Besides these secondary metabolite pathways, membrane transport, cellular community, cell motility and signal transduction may also contribute to the degradation of cigar alkaloids. This genomic information indicated the metabolic potential of different microbes for cigar alkaloids degradation.

## Discussion

Cigar aging is a complex biological and chemical process in which the main role is played by the succession of microbial communities and their metabolic activity. The microbial populations at the beginning of the existing aging phase are derived from colonizing and endophytic microorganisms during the tobacco growing period, and from environmental microorganisms introduced by the fermentation process (e.g., production equipment and environment) (Cai et al. 2021; Han et al. 2016). Although there are condition-mediated changes in the succession and relative abundance of microbial communities during the aging process, the initial microbial population distribution is still one of the most important factors in determining cigar quality, which also provides a theoretical basis for microbial regulation of the aging process.

During the initial stages of the cigar aging process, the fungal community, particularly *Aspergillus*, dominates. These microorganisms possess the ability to secrete amylase and protease enzymes, which rapidly degrade starch and nitrogen sources present in tobacco leaves. The breakdown products, such as reducing sugars and free amino acids, are either utilized by the microorganisms or accumulate in the tobacco. Consequently, the starch and total nitrogen content of the tobacco leaf decrease significantly within the first 30 days of aging. Fungi continue to dominate during this period, while only a few bacteria, such as *Lactobacillus* and *Bacillus*, capable of starch and macromolecular proteolysis, maintain some level of activity, with most of the other bacterial species entering a dormant state. Similar to solid-state fermentation, temperature and humidity play crucial roles in regulating the growth and metabolism of microorganisms during the aging process. As the aging period progresses and humidity decreases, the vitality of fungi gradually declines. Bacteria, on the other hand, can proliferate by utilizing the breakdown products, such as reducing sugars and amino acids, generated through fungal decomposition of larger molecules. The increase in various small molecule flavor compounds is a result of both chemical reactions and the metabolic activities of bacteria, which possess diverse metabolic pathways. Changes in the distribution of non-volatile acids and alterations in the composition of free amino acids mediated by bacteria are slow processes. However, there is potential for artificial regulation using beneficial microorganisms to expedite the aging process and enhance its effectiveness. By manipulating the microbial composition, it may be possible to shorten the aging time and improve the overall quality of the aging process.

The taste and health of cigars both require appropriate levels of starch and alkaloid content. During the aging and fermentation processes, starch is converted into simpler sugars, which can enhance the flavors and aromas (Talhout et al. 2006). The presence of a balanced amount of starch in cigars can contribute to a desirable taste experience by adding depth and complexity to the flavors. However, an excessive amount of starch can result in undesirable flavors and a harsh smoking experience. On the other hand, alkaloids such as nicotine can interact with taste receptors, contribute to the overall taste experience of cigars. Smokers may perceive varying levels of bitterness, richness, and complexity in the flavors of cigars based on the nicotine content. However, excessive nicotine levels can lead to an overpowering taste and potentially unpleasant sensations for individuals who are sensitive to its effects. Moreover, nicotine is a highly addictive substance found in tobacco leaves. Prolonged and excessive nicotine exposure through cigar smoking is associated with numerous health risks. These risks include an increased likelihood of developing cardiovascular diseases, respiratory issues, and certain types of cancer. There are three main methods for the degradation of nicotine: the first method is to use the pyridine pathway in *Pseudomonas* to degrade nicotine. The second method involves using the pyrrolidine pathway in *Pseudomonas putida* to degrade nicotine. The third method involves using the demethylation pathway found in fungi to degrade nicotine. Our study revealed the relationships between microbial communities and alkaloids degradation aging cigar leaves. The compound differences of fermented leaves were closely related to the taxonomic composition and functional profiles of microbial communities. There were more microbial species relating to alkaloids than those relating to starch, reducing sugars and total sugars. Vital species for degradation of starch (*Bacillus pumilus*, *Pseudomonas* sp. 286 and *Aspergillus cristatus*), reducing sugars and total sugars (*Aspergillus cristatus* and *Nitrolancea hollandica*), alkaloids (*Corynespora cassiicola* and *Pseudomonas fulva*) were identified. Therefore, modulating the biodegradation of starch and alkaloids during the aging process can serve as a crucial method for controlling the taste and health of cigars.

## Conclusion

The biodegradation of starch and alkaloid substances in cigars is one of the primary events during the aging process and also has a significant impact on the quality of cigars. The conditions of this process, including temperature, humidity, duration time, and turning frequency, shape the different structural components of cigars by influencing the succession of fungal and bacterial communities. Vital species in the catabolism of starch (*Bacillus pumilus*, *Pseudomonas* sp. 286 and *Aspergillus cristatus*), reducing sugars and total sugars (*Aspergillus cristatus* and *Nitrolancea hollandica*) were identified. *Corynespora cassiicola* and *Pseudomonas fulva* were proposed to contribute to the degradation of nornicotine and neonicotinoid. This study deepens our understanding of the biodegradation mechanisms of starch and alkaloids during the cigar aging process. The identified significant functional microorganisms in this research have important reference value for establishing microbial-based artificial regulation techniques for cigar aging.

## Data Availability

Data and materials described in this study are available from the authors upon reasonable request and availability.
